# Chromosomal analysis of *Physalaemus kroyeri* and *Physalaemus cicada* (Anura, Leptodactylidae)

**DOI:** 10.3897/CompCytogen.v10i2.9319

**Published:** 2016-07-08

**Authors:** Stenio Eder Vittorazzi, Luciana Bolsoni Lourenço, Mirco Solé, Renato Gomes Faria, Shirlei Maria Recco-Pimentel

**Affiliations:** 1 Departamento de Biologia Estrutural e Funcional, Instituto de Biologia, Universidade Estadual de Campinas, 13083-863 Campinas, São Paulo, Brazil; 2 Departamento de Ciências Biológicas, Universidade Estadual de Santa Cruz, 45662-000, Ilhéus, Bahia, Brazil; 3 Departamento de Biologia, Centro de Ciências Biológicas e da Saúde, Universidade Federal de Sergipe, 49100-000, São Cristóvão, Sergipe, Brazil

**Keywords:** NOR, C Banding, PcP190 satDNA

## Abstract

All the species of *Physalaemus* Fitzinger, 1826 karyotyped up until now have been classified as 2n = 22. The species of the *Physalaemus cuvieri* group analyzed by C-banding present a block of heterochromatin in the interstitial region of the short arm of pair 5. *Physalaemus cicada* Bokermann, 1966 has been considered to be a member of the *Physalaemus cuvieri* species group, although its interspecific phylogenetic relationships remain unknown. The PcP190 satellite DNA has been mapped on the chromosomes of most of the species of the *Physalaemus cuvieri* group. For two species, *Physalaemus cicada* and *Physalaemus kroyeri* (Reinhardt & Lütken, 1862), however, only the chromosome number and morphology are known. Given this, the objective of the present study was to analyze the chromosomes of *Physalaemus cicada* and *Physalaemus kroyeri*, primarily by C-banding and PcP190 mapping. The results indicate that *Physalaemus kroyeri* and *Physalaemus cicada* have similar karyotypes, which were typical of *Physalaemus*. In both species, the NORs are located on the long arm of pair 8, and the C-banding indicated that, among other features, *Physalaemus kroyeri* has the interstitial band on chromosome 5, which is however absent in *Physalaemus cicada*. Even so, a number of telomeric bands were observed in *Physalaemus cicada*. The mapping of the PcP190 satellite DNA highlighted areas of the centromeric region of the chromosomes of pair 1 in both species, although in *Physalaemus kroyeri*, heteromorphism was also observed in pair 3. The cytogenetic evidence does not support the inclusion of *Physalaemus cicada* in the *Physalaemus cuvieri* group. In the case of *Physalaemus kroyeri*, the interstitial band on pair 5 is consistent with the existence of a cytogenetic synapomorphy in the *Physalaemus cuvieri* species group.

## Introduction

The family Leptodactylidae is made up of three subfamilies, the Leptodactylinae, Paratelmatobiinae and Leiuperinae (Pyron and Wiens 2011, Fouquet et al. 2013, Frost 2016). The Leiuperinae include five genera, *Edalorhina* Jiménez De La Espada, 1870, *Engystomops* Jiménez De La Espada, 1870, *Physalaemus* Fitzinger, 1826, *Pleurodema* Tschudi, 1838 and *Pseudopaludicola* Miranda-Ribeiro, 1926 (Frost 2016), of which *Physalaemus* is the most diverse, with 47 species (Frost 2016). Based on the phenetic analysis of morphological data, Nascimento et al. (2005) recognized seven groups of *Physalaemus* species, the *Physalaemus cuvieri*, *Physalaemus signifer*, *Physalaemus albifrons*, *Physalaemus deimaticus*, *Physalaemus gracilis*, *Physalaemus henselii* and *Physalaemus olfersii* groups. However, an alternative approach to the phylogeny of these species, based on the analysis of mitochondrial and nuclear data, produced a new proposal, formed by two major clades, *Physalaemus signifer* and *Physalaemus cuvieri*. The *Physalaemus cuvieri* clade encompasses the *Physalaemus cuvieri*, *Physalaemus biligonigerus*, *Physalaemus henselii*, *Physalaemus gracilis* and *Physalaemus olfersii* species groups, as well as the species *Physalaemus aguirrei* Bokermann, 1966 and *Physalaemus cicada* Bokermann, 1966, whose interspecific relationships remain unclear (Lourenço et al. 2015). The *Physalaemus cuvieri* group is the largest of the *Physalaemus cuvieri* clade, formed by nine described species, *Physalaemus cuvieri* Fitzinger, 1826, *Physalaemus albonotatus* (Steindachner, 1864), *Physalaemus centralis* Bokermann, 1962, *Physalaemus cuqui* Lobo, 1993, *Physalaemus ephippifer* (Steindachner, 1864), *Physalaemus erikae* Cruz & Pimenta, 2004, *Physalaemus fischeri* Boulenger, 1890, *Physalaemus kroyeri* (Reinhardt & Lütken, 1862) and *Physalaemus albifrons* (Spix, 1824). In the analysis of Nascimento et al. (2005), *Physalaemus cicada* was included in the *Physalaemus cuvieri* group, although the phylogenetic analyses of Lourenço et al. (2015) did not confirm this asssignment.

All the *Physalaemus* species karyotyped up until the present time show 2n = 22 (Beçak et al. 1970, Denaro 1972, De Lucca et al. 1974, Silva et al. 1999, Silva et al. 2000, Amaral et al. 2000, Lourenço et al. 2006, Ananias et al. 2007, Tomatis et al. 2009, Milani et al. 2010, Nascimento et al. 2010, Provete et al. 2012, Vittorazzi et al. 2014b). The species of the *Physalaemus cuvieri* group studied by C-banding (Silva et al. 1999, Quinderé et al. 2009, Nascimento et al. 2010, Vittorazzi et al. 2014b) all present a block of interstitial heterochromatin in the metacentric chromosome 5, which is a potential cytogenetic marker of the *Physalaemus cuvieri* group (Vittorazzi et al. 2014b, Lourenço et al. 2015).The chromosomal location of the PcP190 satellite DNA is known for *Physalaemus cuvieri*, *Physalaemus centralis*, *Physalaemus albonotatus*, *Physalaemus albifrons* and *Physalaemus ephippifer* (Vittorazzi et al. 2011, Vittorazzi et al. 2014a).

For *Physalaemus cicada* and *Physalaemus kroyeri*, the available cytogenetic data are restricted to the chromosome number and morphology (De Lucca et al. 1974). Given this, the objective of the present study was to evaluate the chromosomal features of these two species, in particular the presence of an interstitial heterochromatic band on chromosome 5, which is recognized as a chromosomal synapomorphy in the *Physalaemus cuvieri* group (Vittorazzi et al. 2014b, Lourenço et al. 2015).

## Material and methods

### Animals

All the individuals belonging to two species included in our analyses were deposited in the Museum of Zoology “Professor Adão José Cardoso” of the Universidade Estadual de Campinas (ZUEC). The sample of *Physalaemus kroyeri* consisted of 13 individuals (Males: ZUEC 17480-17484, 17486-17490, 17492 and 17493; Juveniles: 17485) from the municipality of Ilhéus, in Bahia, Brazil (14°47'46.65"S/ 39°10'19.94"W). For *Physalaemus cicada*, one male specimen (ZUEC 17914) was obtained from Limoeiro, in Pernambuco, Brazil (7°53'31.90"S/35°27'57.41"W) and 13 specimens (Males: ZUEC 20407-2410, 20415, 20419-20422; Females: 20413 and 20417; Juveniles: 20411, 20418) from Poço Redondo in Sergipe, Brazil (9°41'13.14"S/ 37°41'14.95"W).

The animals were collected with permission of the Instituto Brasileiro do Meio Ambiente e dos Recursos Naturais Renováveis (IBAMA/SISBIO – Process number 10678–2, 20336–1 and 33133–1). For the subsequent techniques, all samples were extracted from euthanized specimens using anesthetic application to the skin (5% Lidocaine) to minimize animal suffering, according to recommendations of the Herpetological Animal Care and Use Committee (HACC) of the American Society of Ichthyologists and Herpetologists (available in http://www.asih.org), and approved by SISBIO/Institute Chico Mendes de Conservação da Biodiversidade as a condition for the concession license.

### Chromosome preparation and staining

The metaphases were obtained from intestinal cells of the specimens treated with 2% colchicine for at least 4 hours (following Schmid et al. 2010, or adapted from King and Rofe 1976). The chromosomes were stained with Giemsa (10%) and then C-banded (King 1980). The slides were then processed using the Ag-NOR method (Howell and Black 1980) or stained with DAPI (0.5 μg/mL) or mithramycin (0.5 mg/mL). Chromosomal morphometrics were obtained using the MICROMEASURE v3.3 software (Reeves and Tear 2000) and the classification was based on the criteria of Green and Sessions (1991).

### Extraction, isolation, cloning and sequencing of the DNA

The genomic DNA of *Physalaemus kroyeri* and *Physalaemus cicada* was extracted from samples macerated in TNES buffer (50 mM Tris pH 7.5; 400 mM NaCl; 20 mM EDTA; and 0.5% SDS), following Medeiros et al. (2013). Samples of the genomic DNA of *Physalaemus cicada* and *Physalaemus kroyeri* were submitted to a PCR using the primers P190F (AGA CTG GCT GGG AAT CCC AG) and P190R (AGC TGC TGC GAT CTG ACA AGG) (Vittorazzi et al. 2011) for the isolation of the PcP190 satellite DNA. The resulting sequences were purified and ligated to the pGEM-T Easy vector (Promega, Madison, Wisconsin, USA). The recombinant vectors were used to transform *Escherichia coli* bacteria of the JM109 lineage using a TransformAid™ Bacterial Transformation kit (Fermentas, Burlington, Ontario, Canada), following the maker’s recommendations. The procedures for the selection of the recombinant clones and the extraction of the plasmidial DNA were those proposed by Sambrook et al. (1989).

To sequence the fragments, samples of the amplified PCR products were treated with a BigDye Terminator kit (Applied Biosystems, Foster City, California, USA). After precipitation and drying, the products of this reaction were resuspended in loading dye (1:5 Blue-Dextran-EDTA/Formamide), denatured for 3 minutes at 94°C and analyzed in an ABI 3730XL automatic sequencer.

All the cloned fragments were sequenced, although for the comparative analyses, only the complete PcP190 sequences were used. It is important to note that the partial units were not noticeably different in their composition from the complete sequences.

### Fluorescent *in situ* Hybridization (FISH)

The labeling of the isolated PcP190 satellite DNA probes used in this analysis was based on PCR amplification in the presence of *Digoxigenin*-11-*dUTP* with a DIG Probe Synthesis PCR (Roche, Pensberg, Bavaria, Germany). The probes were mixed with salmon DNA (1 ng/μL of probe) and precipitated with ethanol. All the resulting DNA was dissolved in a hybridization buffer at pH 7 composed of deionized formamide (50%), 2x SSC, phosphate buffer (40 mM), Denhardt’s solution, SDS (1%) and dextran sulfate (10%).

The hybridization method used was that described by Viegas-Péquignot (1992), with adaptations for the detection of the *Digoxigenin*-11-*dUTP*, which was based on the anti-digoxigenin antibody conjugated with rhodamine (Roche, Pensberg, Bavaria, Germany).

## Results

### *Physalaemus kroyeri*

**Figure 1.**
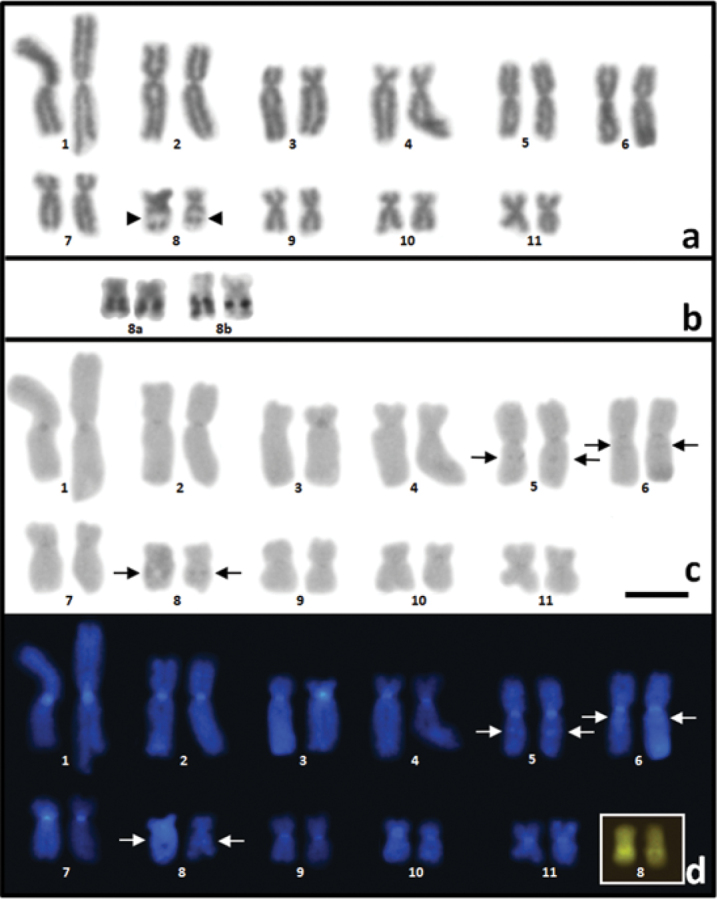
**a** Karyotype of *Physalaemus kroyeri* stained with Giemsa. The arrowhead indicates the secondary constriction **b** Pair 8 showing NOR detected by the Ag-NOR method, in the homozygote (8a) and heterozygote (8b) forms **c** C-banding stained with Giemsa and **d** C-banding stained with DAPI. Highlighted in (**d**), pair 8 stained with mithramycin. In **c** and **d**, the arrows indicate the interstitial heterochromatic bands. Scale bar: 5 μm.

The diploid number of *Physalaemus kroyeri* is 2n = 22, with metacentric pairs 1, 2, 5, 6, 9, and 11, submetacentric pairs 4, 7, 8 and 10, and pair 3 being subtelocentric (Figure 1a; Table 1). A secondary constriction was observed on the long arm of pair 8 (Figure 1a), coinciding with the NOR. In the specimens ZUEC 17480, ZUEC 17481 and ZUEC 17483, the NOR was heteromorphic in size (Figure 1b).

**Table 1.** Morphometry of the karyotypes of *Physalaemus kroyeri* and *Physalaemus cicada*. NC: number of the chromosome; CI: centromeric index; AR: arm ratio; CC: chromosomal classification (Green and Session 1991). A total of 10 karyotypes were analyzed in each species.

*Physalaemus kroyeri*											
NC	1	2	3	4	5	6	7	8	9	10	11
CI	0.45	0.39	0.24	0.26	0.47	0.44	0.32	0.32	0.42	0.34	0.42
AR	1.15	1.5	3.11	2.8	1.11	1.22	2.09	2.01	1.33	1.94	1.34
CC	M	M	ST	SM	M	M	SM	SM	M	SM	M
***Physalaemus cicada***											
NC	1	2	3	4	5	6	7	8	9	10	11
CI	0.45	0.39	0.23	0.27	0.44	0.43	0.3	0.29	0.42	0.37	0.4
AR	1.19	1.51	3.13	2.8	1.23	1.27	2.28	2.43	1.37	1.62	1.48
CC	M	M	ST	SM	M	M	SM	SM	M	M	M

Areas of heterochromatin were detected in the centromeric regions of all the chromosomes, in the pericentromeric region of the long arm of the chromosomes of pair 6, adjacent to the NOR of the chromosomes of pair 8, and interstitially on one of the arms of the metacentric chromosomes of pair 5 (Figure 1c,d). While pair 5 is metacentric, the interstitial C band is located on the arm that appears to be slightly larger. It was also possible to observe a positive mithramycin band together with the NOR (Figure 1d).

### *Physalaemus cicada*

**Figure 2.**
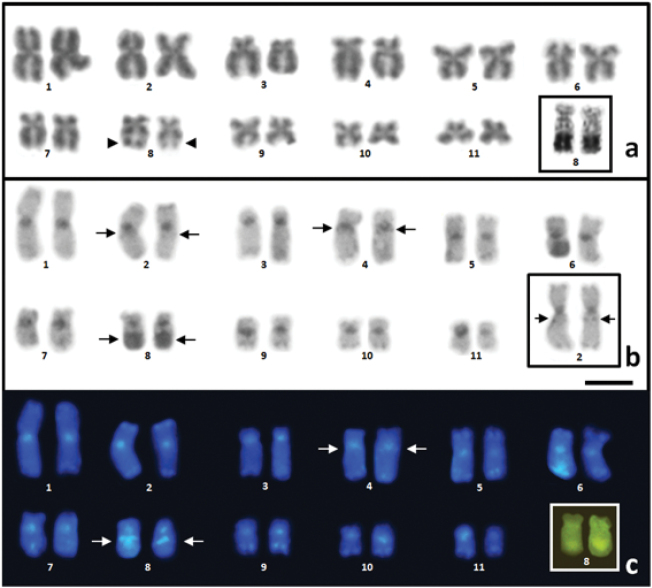
**a** Karyotype of *Physalaemus cicada* stained with Giemsa. The arrowhead indicates the secondary constriction in pair 8, highlighting the NOR in pair 8 **b** C-banding of the karyotype, highlighting the proximal C band in pair 2 **c** C-banding followed by DAPI staining, highlighting pair 8 stained with mithramycin. In **b** and **c**, the arrows indicate the interstitial and pericentromeric heterochromatic bands. Scale bar: 5 μm.

*Physalaemus cicada* has a diploid number of 2n = 22, with metacentric pairs 1, 2, 5, 6, 9, 10 and 11, submetacentric pairs 4, 7 and 8, and one subtelocentric pair, pair 3 (Figure 2a; Table 1). A large secondary constriction can be observed on the long arm of pair 8, together with the NOR (Figure 2a, inset).

Regions of constitutive heterochromatin were detected in the centromeres of all the chromosomes, in the proximal region of the long arm of the chromosomes of pair 2, in the pericentromeric region of the long arm of the chromosomes of pair 4, in the telomeric regions of both arms of the chromosomes of pairs 1, 2, 5, 6 and 7, and a similar pattern, but restricted to the long arms of pairs 3, 4, 9, 10 and 11 (Figure 2b,c). A large block of heterochromatin can be observed on the long arm of pair 8 (Figure 2b), coinciding with the NOR which was also strongly stained by mithramycin in C-banded metaphases (Figure 2c – inset).When C-banding was followed by DAPI staining, all the centromeric and telomeric C-bands were revealed as well as a band adjacent to the NOR (Figure 2).

### PcP190 satellite DNA

After cloning, sequencing, and the search for similar sequences using the BLASTn tool in GenBank, it was possible to conclude that the sequences obtained with the primers P190F and P190R belong to the PcP190 satellite DNA family, which was first identified in *Physalaemus cuvieri* (Vittorazzi et al. 2011).

**Figure 3.**
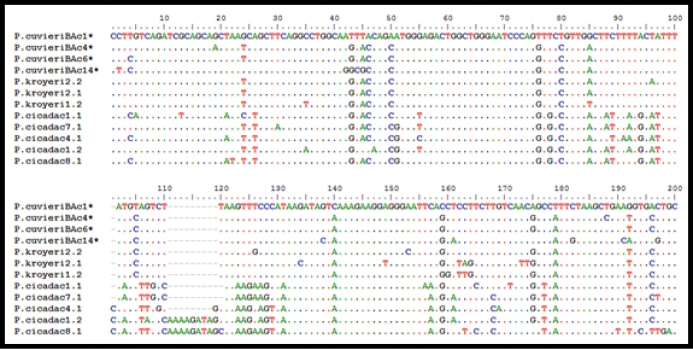
Alignment of the PcP190 satellite DNA sequences of the species *Physalaemus kroyeri*, *Physalaemus cicada* and *Physalaemus cuvieri* available in GenBank* (JF281121, JF281117, JF281109 and JF281124).

It was possible to clone three fragments of the PcP190 satellite DNA of *Physalaemus kroyeri*, all of which contain a complete repeat unit of this satellite DNA, of 190 bps (Figure 3). The mean similarity between these fragments was 95%, and when compared with the PcP190 sequences of *Physalaemus cuvieri* (Vittorazzi et al. 2011), the similarity was 93%. Five complete sequences of the PcP190 were obtained from *Physalaemus cicada*, of which, one was 189 bps in length, two were 192 bps long, and two were 200 bps. These differences in the size of the *Physalaemus cicada* result from a polymorphic region of 20 bps, characterized by substitutions and indels (Figure 3). The mean similarity of the *Physalaemus cicada* sequences was 88%, decreasing to 78% in comparison with *Physalaemus cuvieri* (Vittorazzi et al. 2011). The sequences obtained for *Physalaemus kroyeri* and *Physalaemus cicada* were 79% similar, on average.

**Figure 4.**
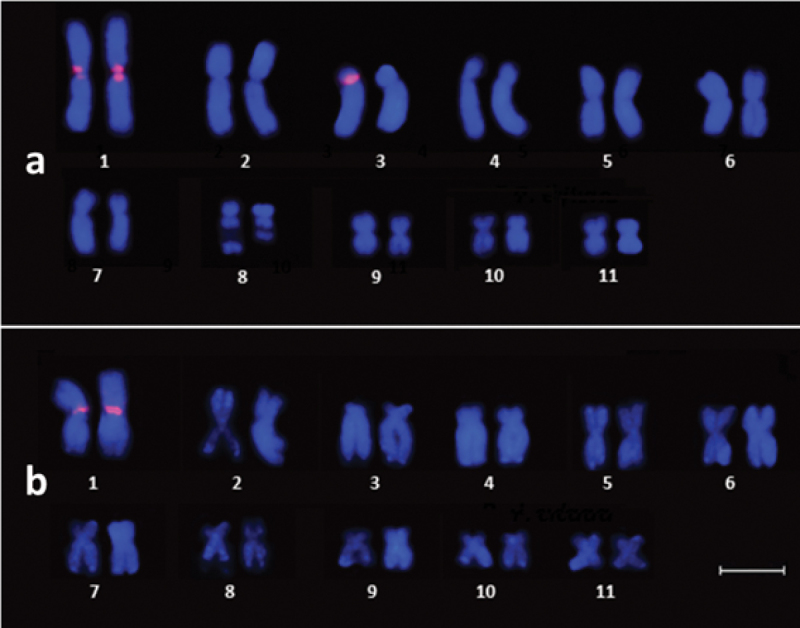
Karyotype of **a** *Physalaemus kroyeri* and **b** *Physalaemus cicada* hybridized with PcP190 satellite DNA probes. Note the signs of hybridization of the probe in the centromeric region of pair 1 in (**a**) and (**b**), and in one of the chromosomes of pair 3 in (**a**). Scale bar: 5 μm.

In the karyotype of *Physalaemus kroyeri*, the PcP190 satellite DNA was detected in the centromeric region of pair 1. In two of the three individuals analyzed, in addition, the PcP190 was also detected in the centromeric region of one of the chromosomes of pair 3 (Figure 4a). In *Physalaemus cicada*, the PcP190 was detected in the centromeric region of pair 1, in the individuals from both Limoeiro and Poço Redondo (Figure 4b).

## Discussion

The number and morphology of the chromosomes observed in the karyotypes of *Physalaemus kroyeri* and *Physalaemus cicada* were the same as those found by De Lucca et al. (1974). The fundamental number (FN) of these karyotypes is 44, which is characteristic of most of the *Physalaemus* species for which cytogenetic data are available, such as *Physalaemus cuvieri* (Beçak et al. 1970, Silva et al. 1999, Quinderé et al. 2009), *Physalaemus soaresi* Izecksohn, 1965 (De Lucca et al. 1974), *Phyllodactylus marmoratus* (Reinhardt & Lütken, 1862) (Beçak et al. 1970, Amaral et al. 2000), *Physalaemus biligonigerus* (Cope, 1861) (Amaral et al. 2000, Silva et al. 2000), *Physalaemus henselii* (Peters, 1872), *Physalaemus riograndensis* Milstead, 1960 (Tomatis et al. 2009), *Physalaemus olfersii* (Lichtenstein & Martens, 1856) (De Lucca et al. 1974; Silva et al. 2000, Milani et al. 2011), *Physalaemus ephippifer* (Nascimento et al. 2010), *Physalaemus barrioi* Bokermann, 1967 (Provete et al. 2012), *Physalaemus albifrons*, *Physalaemus centralis* (Denaro 1972, Vittorazzi et al. 2014b), *Physalaemus albonotatus*, *Physalaemus cuqui*, and *Physalaemus santafecinus* Barrio, 1965 (Vittorazzi et al. 2014b). However, the species of the *Physalaemus signifer* clade and *Physalaemus fernandezae* (Muller, 1926) (part of the *Physalaemus henselii* group of the *Physalaemus cuvieri* clade) (see Lourenço et al. 2015) have FN=42, due to the presence of a telocentric pair classified as pair 11.

Comparing the karyotypes of *Physalaemus kroyeri* and *Physalaemus cicada* with one another and the karyotypes described for other *Physalaemus* species, it is possible to infer homologies in the first seven pairs of chromosomes. This is because the morphology of pairs 1–7 is highly similar in the karyotypes analyzed, despite some differences in size (e.g., *Physalaemus albonotatus* in Vittorazzi et al. 2014b). Even so, it is possible that some of these inferences are erroneous, given that some pairs of chromosomes (pairs 3 and 4, for example, and 5 and 6) are very similar. On the other hand, the recognition of homologies in pairs 8 to 11 is hampered by the fact that these chromosomes are all very small and similar in morphology, except for the telocentric chromosomes classified as pair 11 in the species of the *Physalaemus signifer* clade and *Physalaemus fernandezae* (see Lourenço et al. 2015 and references therein).

In the karyotype of *Physalaemus kroyeri*, the NOR is located interstitially on the long arm of the chromosomes of pair 8, a situation also observed in *Physalaemus albifrons* (Vittorazzi et al. 2014b), which are sister species inferred by Lourenço et al. (2015), and in some populations of *Physalaemus cuvieri* (Quinderé et al. 2009). The results of the present study permit the differentiation of the karyotypes of *Physalaemus albifrons* and *Physalaemus kroyeri* by the presence of interstitial bands of heterochromatin on the long arms of pairs 6 and 8 in *Physalaemus kroyeri*, which are absent in *Physalaemus albifrons* (Vittorazzi et al. 2014b), and an interstitial band on the short arm of pair 8 in *Physalaemus albifrons*, which was absent in *Physalaemus kroyeri*. One other difference between the two species can be observed in pair 1, in which PcP190 satellite DNA is present in *Physalaemus kroyeri*, but not in *Physalaemus albifrons* (Vittorazzi et al. 2014a).

The interstitial C band in the metacentric pair 5 is present in all the species of the *Physalaemus cuvieri* group karyotyped up until now, which Vittorazzi et al. (2014b) proposed as a potential cytogenetic marker for the *Physalaemus cuvieri* group, confirmed by the phylogenetic analysis of Lourenço et al. (2015). The results of the present study also indicate that the marker is present in *Physalaemus kroyeri*, another species of the *Physalaemus cuvieri* group.

While the chromosome pair 5 of *Physalaemus kroyeri* is classified morphologically as metacentric, the arm on which the interstitial band is located is slightly larger, which calls into question the 5p position of this band in the other species of the *Physalaemus cuvieri* group. This difference may have resulted from some structural modification of the chromosome, such as a pericentric inversion, amplification of part of this arm, or a deletion on the opposite arm. Whatever the case, the difference in the position of this interstitial band does not alter its status as a chromosomal synapomorphy in the *Physalaemus cuvieri* group.

The absence of this interstitial band of heterochromatin on chromosome 5 in the karyotype of *Physalaemus cicada*, keeps the interpecific relationships of this species in doubt. While *Physalaemus cicada* has been considered to be a member of the *Physalaemus cuvieri* group, based on its morphological similarities (Lynch 1970, Nascimento et al. 2005), Lourenço et al. (2015) found no support for this arrangement in their phylogenetic analyses.

### PcP190 satellite DNA

It was possible to recognize PcP190 satellite DNA in both *Physalaemus kroyeri* and *Physalaemus cicada*, as found in a number of other *Physalaemus* species, such as *Physalaemus cuvieri*, *Physalaemus centralis*, *Physalaemus albonotatus*, *Physalaemus albifrons*, *Physalaemus ephippifer*, *Phyllodactylus marmoratus* and *Physalaemus nattereri* (Steindachner, 1863), as well as members of other leptodactylid genera, such as *Pleurodema diplolister* (Peters, 1870), *Leptodactylus latrans* (Steffen, 1815) and *Crossodactylus gaudichaudii* Duméril & Bibron, 1841 (Vittorazzi et al. 2014a) and in the hylid genus Pseudis (Gatto et al. 2016). This sequence is well conserved, and appears to have an ancient origin in the anurans (Vittorazzi et al. 2014a, Gatto et al. 2016).

In *Physalaemus cicada*, both the sequences and the location of the PcP190 in the karyotype provide interesting insights into the comparison of this species with those of the *Physalaemus cuvieri* group. On average, the PcP190 of the species of this group are 90% similar to one another (Vittorazzi et al. 2014a), although this falls to 78% in the comparison with *Physalaemus cicada*. The chromosomal mapping of these sequences in *Physalaemus cicada* is also distinct from that of the *Physalaemus cuvieri* group, given the lack of a PcP190 site in pair 3, which is characteristic of all the species of the *Physalaemus cuvieri* group analyzed to date (Vittorazzi et al. 2011, 2014a). These differences may reflect a more distant phylogenetic relationship between *Physalaemus cicada* and the species of the *Physalaemus cuvieri* group. However, we must consider that given family of satellite DNA may present a different number of repetitions, even in closely-related species, given that the evolutionary dynamics of these sequences favors their continuous amplification and deletion in the genome. This is covered in the original proposal for a DNA satellite library (Fry and Salser 1977, Meštrović et al. 1998), which indicated that different families of satellite DNA coexist in a genome, but that new families may arise continually through the restructuring of the distribution and quantity of the older sequences.

## Conclusion

The interstitial heterochromatic band on the metacentric chromosome 5, considered to be a cytogenetic synapomorphy of the *Physalaemus cuvieri* species group was found in *Physalaemus kroyeri*. In contrast, this marker was absent in *Physalaemus cicada*, which did not support the inclusion of *Physalaemus cicada* in the *Physalaemus cuvieri* species group.

## Contribution of the authors

SEV developed the study, collected *Physalaemus kroyeri* and *Physalaemus cicada*, ran the analyses and drafted the manuscript. MS collected *Physalaemus kroyeri* and RGF collected *Physalaemus cicada*, both these authors revised the manuscript. SMRP and LBL developed and coordinated the study and revised the manuscript.

## References

[B1] Amaral MJLV Cardoso AJ Recco-Pimentel SM (2000) Cytogenetic analysis of three *Physalaemus* species (Amphibia, Anura). Caryologia 53: 283–288. doi: 10.1080/00087114.2000.10589207

[B2] Ananias F Bombeiro AL Silva CDB Silva APZ Haddad CFB (2007) Cytogenetics of *Eupemphix nattereri* Steindachner, 1863 (Anura: Leiuperidae) and karyotypic similarity with species of related genera: taxonomic implications. Acta Zoologica Sinica 53: 285–293.

[B3] Beçak ML Denaro L Beçak W (1970) Polyploidy and mechanisms of karyotypic diversification in Amphibia. Cytogenetics 9: 225–238. doi: 10.1159/000130093 5474808 10.1159/000130093

[B4] De Lucca EJ Jim J Foresti F (1974) Chromosomal studies in twelve species of Leptodactylidae and one Brachycephalidae. Caryologia 27: 183–191. doi: 10.1080/00087114.1974.10796573

[B5] Denaro L (1972) Karyotypes of Leptodactylidae Anurans. Journal of Herpetology 6: 71–74. doi: 10.2307/1563095

[B6] Frost DR (2016) Amphibians species of the world: an online reference. Version 6.0 (15 march 2016). American Museum of Natural History, New York http://research.amnh.org/herpetology/amphibia/index.html

[B7] Fry K Salser W (1977) Nucleotide sequences of HS-α satellite DNA from kangaroo rat *Dipodomys ordii* and characterization of similar sequences in other rodents. Cell 12: 1069–1084. doi: 10.1016/0092-8674(77)90170-2 597857 10.1016/0092-8674(77)90170-2

[B8] Fouquet A Blotto BL Maronna MM Verdade VK Juncá FA de Sá R Rodrigues MT (2013) Unexpected phylogenetic positions of the genera *Rupirana* and *Crossodactylodes* reveal insights into the biogeography and reproductive evolution of leptodactylid frogs. Molecular Phylogenetics and Evolution 67: 445–457. doi: 10.1016/j.ympev.2013.02.009 23454092 10.1016/j.ympev.2013.02.009

[B9] Gatto KP Busin CS Lourenço LB (2016) Unraveling the sex chromosome heteromorphism of the paradoxical frog *Pseudis tocantins*. PLoS ONE 11(5): . doi: 10.1371/journal.pone.0156176 10.1371/journal.pone.0156176 PMC4877019 27214234

[B10] Green DM Sessions SK (1991) Nomenclature for Chromosomes. In: Amphibian cytogenetics and evolution. Academic Press, San Diego, 431–432. doi: 10.1016/b978-0-12-297880-7.50021-4

[B11] Howell WM Black DA (1980) Controlled silver staining of nucleolus organizer regions with a protective colloidal developer: a 1-step method. Experientia 36: 1014–1015. doi: 10.1007/BF01953855 6160049 10.1007/BF01953855

[B12] King M (1980) C-banding studies on Australian hylid frogs: secondary constriction structure and the concept of euchromatin transformation. Chromosoma 80: 191–217. doi: 10.1007/BF00286300

[B13] King M Rofe R (1976) Karyotypic variation in the Australian gekko *Phyllodactylus marmoratus* (Gray) (Gekkonidae: Reptilia). Chromosoma 54: 75–87. doi: 10.1007/BF00331835 1248335 10.1007/BF00331835

[B14] Lourenço LB Nascimento JAA Andrade GV Rossa-Feres DC Recco-Pimentel SM (2006) Chromosomal analyses of the leptodactylids *Pleurodema diplolistris* and *Physalaemus nattereri* (Amphibia, Anura). Amphibia-Reptilia 27: 481–489. doi: 10.1163/156853806778877103

[B15] Lourenço LB Targueta CP Baldo D Nascimento J Garcia PCA Andrade GV Haddad CFB Recco-Pimentel SM (2015) Phylogeny of frogs from the genus *Physalaemus* (Anura, Leptodactylidae) inferred from mitochondrial and nuclear gene sequences. Molecular Phylogenetics and Evolution 92: 204–216. doi: 10.1016/j.ympev.2015.06.011 26143292 10.1016/j.ympev.2015.06.011

[B16] Lynch JD (1970) Systematic status of the American leptodactylid frog genera *Engystomops*, *Eupemphix*, and *Physalaemus*. Copeia 1970: 488–496. doi: 10.2307/1442276

[B17] Medeiros LR Lourenço LB Rossa-Feres DC Lima AP Andrade GV Giaretta A Egito GTB Recco-Pimentel SM (2013) Comparative cytogenetic analysis of some species of the *Dendropsophus microcephalus* group (Anura, Hylidae) in the light of phylogenetic inferences. BMC Genetics 14: . doi: 10.1186/1471-2156-14-59 10.1186/1471-2156-14-59 PMC3710474 23822759

[B18] Meštrović N Plohl M Mravinac B Ugarković ĐÐ (1998) Evolution of satellite DNAs from the genus *Palorus* - experimental evidence for the ‘library’ hypothesis. Molecular and Biology Evolution 15: 1062–1068. doi: 10.1093/oxfordjournals.molbev.a026005 10.1093/oxfordjournals.molbev.a026005 9718733

[B19] Milani M Cassini CS Recco-Pimentel SM Lourenço LB (2010) Karyotypic data detect interpopulational variation in *Physalaemus olfersii* and the first case of a supernumerary chromosome in the genus. Animal Biology Journal 2: 21–28. doi: 10.1655/HERPETOLOGICA-D-12-00020

[B20] Nascimento J Quinderé YRSD Recco-Pimentel SM Lima JRF Lourenço LB (2010) Heteromorphic Z and W sex chromosomes in *Physalaemus ephippifer* (Steindachner,1864) (Anura, Leiuperidae). Genetica 138: 1127–1132. doi: 10.1007/s10709-010-9501-9 20882322 10.1007/s10709-010-9501-9

[B21] Nascimento LB Caramaschi U Cruz CAG (2005) Taxonomic review of the species groups of the genus *Physalaemus* Fitzinger, 1826 with revalidation of the genera *Engystomops* Jiménez-de-La-Espada, 1872 and *Eupemphix* Steindachner, 1863 (Amphibia, Anura, Leptodactylidae). Arquivos do Museu Nacional Rio de Janeiro 63: 297–320.

[B22] Provete DB Garey MV Toledo LF Nascimento J Lourenço LB Rossa-Feres DC Haddad CFB (2012) Redescription of *Physalaemus barrioi* (Anura: Leiuperidae). Copeia 2012: 507–518. doi: 10.1643/CH-10-142

[B23] Pyron RA Wiens JJ (2011) A large-scale phylogeny of Amphibia including over 2,800 species, and a revised classification of extant frogs, salamanders, and caecilians. Molecular Phylogenetics and Evolution 61: 543–583. doi: 10.1016/j.ympev.2011.06.012 21723399 10.1016/j.ympev.2011.06.012

[B24] Quinderé YRD Lourenço LB Andrade GV Tomatis C Baldo D Recco-Pimentel SM (2009) Polytypic and polymorphic NOR variations in the widespread anuran *Physalaemus cuvieri* (Anura, Leiuperidae). Biological Research 42: 79–92. doi: 10.4067/S0716-97602009000100008 19621135

[B25] Reeves A Tear J (2000) MicroMeasure for Windows, version 3.3. Free program distributed by the authors over the Internet from http://www.colostate.edu/Depts/Biology/MicroMeasure.

[B26] Sambrook J Fritsch EF Maniatis T (1989) Molecular Cloning – A Laboratory Manual. Cold Spring Harbor Laboratory Press, Cold Spring Harbor.

[B27] Schmid M Bogart JP Hedges SB (2010) The chromosomes of terraranan frogs. Insights into vertebrate cytogenetics. Cytogenetic and Genome Research 130–131: 1–568. doi: 10.1159/000301339 10.1159/000301339 21063086

[B28] Silva APZ Baldissera-Jr FA Haddad CFB Kasahara S (2000) Karyotypes and nucleolus organizer regions of the genus *Physalaemus* (Anura, Leptodactylidae). Iheringia, Série Zoológica 88: 159–164.

[B29] Silva APZ Haddad CFB Kasahara S (1999) Nucleolus organizer regions in *Physalaemus cuvieri* (Anura, Leptodactylidae), with evidence of a unique case of Ag-NOR variability. Hereditas 131: 135–141. doi: 10.1111/j.1601-5223.1999.00135.x 10712096 10.1111/j.1601-5223.1999.00135.x

[B30] Tomatis CG Baldo D Kolenc F Borteiro C (2009) Chromosomal variation in the species of the *Physalaemus henselii* group (Anura, Leiuperidae). Journal of Herpetology 43: 555–560. doi: 10.1670/08-122R1.1

[B31] Viegas-Pequignot (1992) In situ hybridization to chromosomes with biotinylated probes. In: Willernson D (Ed.) In situ hybridization: a practical approach. Oxford University Press-IRL Press, Oxford, 137–158.

[B32] Vittorazzi SE Lourenço LB Del-Grande ML Recco-Pimentel SM (2011) Satellite DNA derived from 5S rDNA in *Physalaemus cuvieri* (Anura, Leiuperidae). Cytogenetic and Genome Research 134: 101–107. doi: 10.1159/000325540 21464559 10.1159/000325540

[B33] Vittorazzi SE Lourenço LB Recco-Pimentel SM (2014a) Long-time evolution and highly dynamic satellite DNA in leptodactylid and hylodid frogs. BMC Genetics 15: . doi: 10.1186/s12863-014-0111-x 10.1186/s12863-014-0111-x PMC4201667 25316286

[B34] Vittorazzi SE Quinderé YRSD Recco-Pimentel SM Tomatis C Baldo D Lima JRF Ferro JM Lima JD Lourenço LB (2014b) Comparative cytogenetics of *Physalaemus albifrons* and *Physalaemus cuvieri* species groups (Anura, Leptodactylidae). Comparative Cytogenetics 8: 103–123. doi: 10.3897/CompCytogen.v8i2.6414 25147623 10.3897/CompCytogen.v8i2.6414 PMC4137282

